# Sequence variation in telomerase reverse transcriptase (*TERT*) as a determinant of risk of cardiovascular disease: the Atherosclerosis Risk in Communities (ARIC) study

**DOI:** 10.1186/s12881-015-0194-x

**Published:** 2015-07-23

**Authors:** Jan Bressler, Nora Franceschini, Ellen W. Demerath, Thomas H. Mosley, Aaron R. Folsom, Eric Boerwinkle

**Affiliations:** Human Genetics Center, School of Public Health, University of Texas Health Science Center at Houston, 1200 Pressler Street, Houston, TX 77030 USA; Department of Epidemiology, University of North Carolina Gillings School of Global Public Health, 135 Dauer Drive, Chapel Hill, NC 27599 USA; Division of Epidemiology and Community Health, University of Minnesota School of Public Health, 1300 South Second Street, Minneapolis, MN 55454 USA; Department of Medicine, Division of Geriatrics, University of Mississippi Medical Center, 2500 North State Street, Jackson, MS 39216 USA; Human Genome Sequencing Center, Baylor College of Medicine, 1 Baylor Plaza, Houston, TX 77030 USA

**Keywords:** Genetic epidemiology, Myocardial infarction, Cerebrovascular stroke, Telomere homeostasis, Cell senescence, Cellular aging

## Abstract

**Background:**

Telomerase reverse transcriptase (TERT) maintains telomere ends during DNA replication by catalyzing the addition of short telomere repeats. The expression of telomerase is normally repressed in somatic cells leading to a gradual shortening of telomeres and cellular senescence with aging. Interindividual variation in leukocyte telomere length has been previously associated with susceptibility to cardiovascular disease. The aim of the present study was to determine whether six variants in the *TERT* gene are associated with risk of incident coronary heart disease, incident ischemic stroke, and mortality in participants in the biracial population-based Atherosclerosis Risk in Communities (ARIC) study, including rs2736100 that was found to influence mean telomere length in a genome-wide analysis.

**Methods:**

ARIC is a prospective study of the etiology and natural history of atherosclerosis in 15,792 individuals aged 45 to 64 years at baseline in 1987–1989. Haplotype tagging SNPs in *TERT* were genotyped using a custom array containing nearly 49,000 SNPs in 2,100 genes associated with cardiovascular and metabolic phenotypes. Cox proportional hazards models were used to assess the association between the *TERT* polymorphisms and incident cardiovascular disease and mortality over a 20-year follow-up period in 8,907 whites and 3,022 African-Americans with no history of disease at the baseline examination, while individuals with prevalent cardiovascular disease were not excluded from the analyses of mortality.

**Results:**

After adjustment for age and gender, and assuming an additive genetic model, rs2736122 and rs2853668 were nominally associated with incident coronary heart disease (hazards rate ratio = 1.20, p = 0.02, 95 % confidence interval = 1.03– 1.40) and stroke (hazards rate ratio = 1.17, p = 0.05, 95 % confidence interval = 1.00 - 1.38), respectively, in African-Americans. None of the variants was significantly associated with cardiovascular disease in white study participants or with mortality in either racial group.

**Conclusions:**

Replication in additional population-based samples combined with genotyping of polymorphisms in other genes involved in maintenance of telomere length may help to determine whether genetic variants associated with telomere homeostasis influence the risk of cardiovascular disease in middle-aged adults.

## Background

Telomeres are DNA-protein complexes that protect the ends of chromosomes. Telomerase maintains telomere ends during DNA replication by catalyzing the addition of short telomere repeats (TTAGGG). The enzyme is comprised of a protein with reverse transcriptase activity that is encoded by the telomerase reverse transcriptase (*TERT*) gene, and a telomerase RNA component (TERC) which serves as a template for the telomere repeat after recognition of a single stranded G-rich primer [1]. Expression of telomerase is normally repressed in somatic cells leading to a gradual shortening of telomeres and cellular senescence with aging [2, 3]. Heritability of telomere length in humans has been reported to range from 36 % - 90 % [4, 5].

Leukocyte telomere length has been reported to be associated with susceptibility to cardiovascular disease [6]. When mean telomere length was measured in 10 patients with severe coronary artery disease and compared to that observed for 20 controls, the size was significantly reduced and equivalent to that found in individuals without heart disease who were 9 years older [7]. Significantly shorter telomeres were also detected in leukocyte DNA from 203 subjects who had had a myocardial infarction (MI) before the age of 50 [8], from 620 chronic heart failure patients [9], and from 150 stroke patients [10] when compared to controls. Shorter telomere length has also been reported to be associated with a higher prevalence of both atherothrombotic and hemorrhagic stroke in a Chinese case–control study [11]. Finally, in four prospective studies that evaluated disease incidence, there was an increased risk of coronary artery disease [12], MI [13–15], and stroke [13] associated with shorter telomere length, while no association was found with ischemic stroke in either the Nurses’ Health Study or the Physicians’ Health Study [16, 17]. Taken together, these results suggest that variation in telomere length may play a role in the risk and progression of cardiovascular disease. An association between survival and leukocyte telomere length has also been observed in several previous epidemiological studies [18–20].

Recently, a single nucleotide polymorphism (SNP) (rs2736100) located within an intron of *TERT* was significantly associated with mean leukocyte telomere length in a genome-wide association (GWA) study in which 37,684 individuals from fifteen cohorts were included in the discovery set. The estimated per-allele effect of addition of the A allele was 94.2 base pairs, equivalent to 3.14 years of age-related telomere shortening [21]. The aim of the present study was to determine whether six haplotype tagging SNPs located within the *TERT* gene or the 5’ promoter region including rs2736100 were associated with risk of incident coronary heart disease (CHD), ischemic stroke, and all-cause mortality in participants in the large biracial population-based ARIC cohort. To date, there have been few previous investigations of the role of telomerase variants in cardiovascular disease and its risk factors that have included individuals of African ancestry [22].

## Methods

### Atherosclerosis Risk in Communities (ARIC) Study

The ARIC study was designed to study the development of atherosclerosis in 15,792 individuals aged 45–64 years. At the time of recruitment in 1987–1989, the participants resided in Forsyth County, North Carolina; Jackson, Mississippi (African-Americans only); northwestern suburbs of Minneapolis, Minnesota; or Washington County, Maryland and were selected by probability sampling. Incident cardiovascular events were ascertained by annual telephone contact, and surveillance of local hospital discharge lists and death records from state vital statistics offices. CHD cases were defined as either fatal CHD or a definite or probable MI. Definite and probable stroke were defined as a rapid focal neurological deficit lasting 24 hours or until death; validation of stroke hospitalization has been described elsewhere [23]. In brief, records for eligible hospitalizations were abstracted by a single trained nurse and classified by a standardized computer algorithm, and were also reviewed by a trained physician. Any disagreements between the computer diagnosis and that of the reviewing physician were adjudicated by a second physician. Incident CHD, ischemic stroke, and death in this study included events from 1987 through December 31, 2011. Individuals were excluded from all of the analyses if they were neither African-American nor white (n = 48); if they were African-Americans from the Minnesota or Maryland field centers (n = 55) due to the small numbers recruited from these sites; or if they did not consent to use or storage of their DNA (n = 44). Participants with prevalent CHD, stroke, or transient ischemic attack were excluded from the analyses of incident CHD and ischemic stroke (n = 1,430) as were those with missing genotype data for all sequence variants (n = 2,206). The final study sample at risk of cardiovascular disease consisted of 8,987 white and 3,022 African-American study participants. Subjects with prevalent CHD or stroke were not excluded from the analyses of all-cause mortality. The study design and methods were approved by the institutional review boards at the collaborating medical centers: University of Mississippi Medical Center Institutional Review Board (Jackson Field Center); Wake Forest University Health Sciences Institutional Review Board (Forsyth County Field Center); University of Minnesota Institutional Review Board (Minnesota Field Center); and the Johns Hopkins School of Public Health Institutional Review Board (Washington County Field Center). Informed consent was provided in writing. A detailed description of the ARIC study has been published previously [24].

### *TERT* polymorphisms and genotyping

Six SNPs either within the *TERT* gene or the 5-kb proximal promoter region were genotyped as previously described [25]. The *TERT* SNPs were among a panel of nearly 49,000 SNPs in 2,100 genes associated with cardiovascular and metabolic phenotypes selected for inclusion on the custom Illumina IBC (ITMAT-Broad-CARe) array as part of the shared Candidate Gene Association Resource (CARe) funded by the National Heart, Lung, and Blood Institute [26]. Systematic searching of the PubMed citation database (http://www.ncbi.nlm.nih.gov/pubmed), pathway-based bioinformatics tools, and advance access to the results of findings from GWA studies of diabetes, hypertension, and coronary artery disease were used to develop an initial list of genes that were then prioritized by investigators from the nine cohorts participating in the CARe Consortium. Genes and pathways implicated in cardiovascular disease as well as lipid metabolism, thrombogenesis, insulin resistance, metabolism, inflammation, oxidative stress, and apoptosis were of particular interest in the selection process. For most of the genes on the array including *TERT* (n = 1,784), haplotype tagging SNPs were selected to capture genetic variation represented in the four HapMap populations [27] and SeattleSNPs [28] resequencing project. Three *TERT* variants present on the array were excluded from further analysis either because they were monomorphic or did not meet Hardy-Weinberg equilibrium expectations in both whites and African-Americans.

### Clinical and laboratory measurements

The clinical and laboratory measurements used for this study were assessed during the first clinical examination in 1987–1989 and have been described previously [29, 30]. Plasma total cholesterol and triglycerides were measured by enzymatic methods and low density lipoprotein (LDL) cholesterol was calculated [31]. High density lipoprotein (HDL) cholesterol was measured after dextran-magnesium precipitation of non-HDL [32]. Blood pressure was measured three times while seated using a random-zero sphygmomanometer and the last two measurements were averaged for analysis. Individuals with diastolic blood pressure ≥ 90 mm Hg, systolic blood pressure ≥140 mm Hg, or who used antihypertensive medication were defined as having hypertension. Fasting serum glucose was measured by a standard hexokinase method on a Coulter DACOS chemistry analyzer (Coulter Instruments, Fullerton, CA). The case definition for diabetes was a fasting glucose level > 7.0 mmol/L, a nonfasting glucose level >11.1 mmol/L, and/or self-reported physician diagnosis or treatment for diabetes. Body weight and other anthropometric variables were measured by trained technicians according to standardized protocols. Body mass index (BMI) was calculated as weight in kilograms/(height in meters)^2^. Information on cigarette smoking and alcohol consumption was obtained using an interviewer-administered questionnaire, and both smoking and drinking status was classified as current, former, or never.

### Statistical analysis

Hardy-Weinberg equilibrium was tested for each SNP separately by race using a χ^2^ goodness-of-fit test prior to the application of any exclusion criteria. Linkage disequilibrium (LD) was estimated using Haploview version 4.2 [33]. Proportions, mean values, and standard deviations were calculated for clinical and demographic variables relevant to cardiovascular disease. Comparisons between groups were performed using chi square tests for categorical variables and t-tests for continuous variables. Cox proportional hazards models were used to estimate hazard rate ratios (HRR) for incident CHD and ischemic stroke, and for death from all causes for each addition of the minor allele for each SNP. The genotypes for rs2736100 were coded in both races with respect to the allele previously shown to be associated with shorter telomere length [21]. Analyses of rs6863494 were only carried out for African-American study participants since this variant was monomorphic in whites. Regression models were adjusted for either age and gender (model 1), or for age, gender, and a panel of established cardiovascular risk factors including BMI, current smoking, diabetes, hypertension, and HDL and LDL cholesterol (model 2). The proportional hazards assumption was met for all of the *TERT* SNPs tested individually by race with the exception of rs2736122 (model 1) and rs4246742 (models 1 and 2) when analyzed in whites for association with incident CHD, and rs2853668 (model 1) in the analyses of mortality in whites [34]. In the analyses of CHD and ischemic stroke, follow-up time was calculated from the date of the baseline visit to the date of the first event. For the non-cases, follow-up continued through the date of last contact, or the date of death if the date of last contact had occurred within one year. In the analyses of all-cause mortality, follow-up continued through either the date of death or December 31, 2011. A two-sided p-value of 0.05 was considered statistically significant, and the Bonferroni correction was used to adjust for multiple comparisons. The results are presented separately by self-reported racial group. Power calculations were performed using the Cox regression module of the Power Analysis and Sample Size computer program [35]. Using the observed incidence of CHD and ischemic stroke in each racial group, the allele frequency for each *TERT* polymorphism in African-Americans and whites, and a Bonferroni corrected p-value of 0.002 (0.05/6 variants x 2 phenotypes x 2 races), there was greater than 90 % statistical power to detect a HRR of ≥ 1.1 for each *TERT* variant. All of the statistical analyses were performed using Stata version 9.0 (Stata Corporation, College Station, TX).

## Results

The allele and genotype frequencies for six *TERT* polymorphisms evaluated in this study (Table 1) were in accordance with Hardy-Weinberg expectations for both white and African-American study subjects (all p > 0.05). When LD was estimated for these variants, the SNPs were not highly correlated for either white or African-American study participants (all r^2^ < 0.15) (Table 2). A description of the study sample at the first clinical visit stratified by race is shown in Table 3. There were 403 incident CHD cases (13.3 %) and 287 ischemic stroke cases (9.5 %) ascertained in African-American subjects during an average follow-up period of 20.0 years, and 933 CHD cases (10.4 %) and 452 stroke cases (5.0 %) identified in whites during an average follow-up period of 20.4 years. All of the clinical and demographic characteristics differed significantly between white and African-American participants with the exception of the levels of total and LDL cholesterol.

**Table 1:** *TERT* genotype and allele frequencies stratified by race. ARIC study (1987–1989)

	African-American		MAF	White		MAF	p
dbSNP ID	N %			N %			
rs2736122							
GG	1,857	56.1	0.25	5,283	53.8	0.27	0.04
AG	1,246	37.7		3,835	39.1		
AA	205	6.2		698	7.1		
rs4246742							
TT	1,444	43.5	0.35	7,037	71.6	0.15	<0.01
AT	1,453	43.8		2,549	25.9		
AA	421	12.7		241	2.5		
rs6863494							
TT	2,950	90.3	0.05	9,823	100..0	0.00	<0.01
CT	305	9.3		2	0.0		
CC	13	0.4		0	0.0		
rs4975605							
CC	1,012	30.6	0.45	2,742	27.9	0.47	<0.01
AC	1,619	48.9		4,861	49.5		
AA	679	20.5		2,218	22.6		
rs2736100*							
CC	683	20.6	0.54	2,533	25.8	0.49	<0.01
CA	1,661	50.0		4,932	50.2		
AA	975	29.4		2,361	24.0		
rs2853668							
GG	841	25.3	0.50	5,432	55.3	0.26	<0.01
TG	1,647	49.6		3,707	37.7		
TT	831	25.1		688	7.0		

dbSNP, The National Center for Biotechnology Information’s SNP database; SNP, single nucleotide polymorphism; ID, identification; N, number; MAF, minor allele frequency; p, p-value for difference in genotype frequencies between racial groups evaluated by Pearson’s chi-squared test; *A allele previously associated with shorter telomere length [21]

**Table 2:** Linkage disequilibrium between *TERT* single nucleotide polymorphisms

LD	*TERT* SNP						
r^2^, White		rs2736122	rs4246742	rs6863494*	rs4975605	rs2736100	rs2853668
	rs2736122	x	0.053	---	0.062	0.058	0.021
	rs4246742	0.053	x	---	0.09	0.005	0.006
	rs6863494			x			
	rs4975605	0.062	0.09	---	x	0.04	0.034
	rs2736100	0.058	0.005	---	0.04	x	0.13
	rs2853668	0.021	0.006	---	0.034	0.13	x
r^2^, African-American		rs2736122	rs4246742	rs6863494	rs4975605	rs2736100	rs2853668
	rs2736122	x	0.001	0.011	0.052	0.001	0.016
	rs4246742	0.001	x	0.097	0.047	0.015	0.001
	rs6863494	0.011	0.097	x	0.032	0.024	0.004
	rs4975605	0.052	0.047	0.032	x	0.021	0.011
	rs2736100	0.001	0.015	0.024	0.021	x	0.023
	rs2853668	0.016	0.001	0.004	0.011	0.023	x

LD, linkage disequilibrium; SNP, single nucleotide polymorphism; *rs6863494 is monomorphic in whites

**Table 3:** Race-specific clinical and demographic characteristics. ARIC participants free of CVD (1987 – 1989)

	N	AA	N	White	p
		(N = 3,022)		(N = 8,987)	
		N (%)		N (%)	
Male	3,022	1,081 (35.8)	8,987	4,030 (44.8)	<0.001
Current smokers	3,020	857 (28.4)	8,984	2,145 (23.9)	<0.001
Current alcohol	2,994	951 (31.8)	8,975	5,909 (65.8)	<0.001
Hypertension	3,009	1,613 (53.6)	8,953	2,250 (25.1)	<0.001
Diabetes	2,953	532 (18.0)	8,972	715 (8.0)	<0.001
Incident MI/Fatal CHD	3,022	403 (13.3)	8,987	933 (10.4)	<0.001
Incident ischemic stroke	3,022	287 (9.5)	8,987	452 (5.0)	<0.001
		Mean (SD)		Mean (SD)	
Age (years)	3,022	53.1 (5.7)	8,987	54.1 (5.7)	<0.001
DBP, mm Hg	3,022	79.6 (11.8)	8,983	71.6 (10.0)	<0.001
SBP, mm Hg	3,022	127.8 (20.3)	8,984	118.2 (16.9)	<0.001
Glucose (mmol/L)	2,941	6.4 (3.0)	8,980	5.8 (1.6)	<0.001
Insulin (pmol/L)	2,941	138.0 (291.4)	8,979	81.4 (94.9)	<0.001
BMI (kg/m^2^)	3,019	29.7 (6.1)	8,980	26.9 (4.8)	<0.001
Total cholesterol, mmol/L	2,895	5.6 (1.2)	8,971	5.5 (1.0)	0.503
LDL cholesterol, mmol/L	2,870	3.6 (1.1)	8,832	3.5 (1.0)	0.281
HDL cholesterol, mmol/L	2,895	1.4 (0.4)	8,973	1.3 (0.4)	<0.001
Triglycerides, mmol/L	2,896	1.3 (0.9)	8,973	1.5 (1.0)	<0.001

CVD, cardiovascular disease; N, number; AA, African-American; p, p-value for tests of differences of group means determined by t-tests or of categorical values evaluated by Pearson’s chi-squared test between racial groups; MI, myocardial infarction; CHD, coronary heart disease; DBP, diastolic blood pressure; SBP, systolic blood pressure; BMI, body mass index; LDL, low density lipoprotein; HDL, high density lipoprotein

The results of the analysis of the association between the *TERT* sequence variants and incident CHD and ischemic stroke are displayed in Tables 4 and 5, respectively. SNP rs2736122 was nominally associated with incident CHD in African-Americans both in the minimally adjusted Cox regression model (HRR = 1.20, p = 0.02, 95 % confidence interval (CI) = 1.03 – 1.40) and in a second model that was further adjusted for a panel of established cardiovascular risk factors (HRR = 1.18, p = 0.04, 95 % CI = 1.01 – 1.39). Similarly, one of the genetic variants was nominally associated with incident ischemic stroke (rs2853668) in African-Americans in a model adjusted for age and gender (HRR = 1.17, p = 0.05, 95 % CI = 1.00 – 1.38), but this relationship was attenuated after BMI, current smoking, and diabetes and hypertension case status were added to the regression models. There were also 1,203 (36.2 %) and 2,875 deaths (29.3 %) among African-American and white participants, respectively, during the mean 20.5-year follow-up period. All-cause mortality was assessed but no association with any of the *TERT* sequence variants was found for either racial group (all p > 0.15) (Table 6). None of the associations described above remained significant after correction for multiple comparisons.

**Table 4:** *TERT* sequence variation and incident coronary heart disease. ARIC study (1987 – 2011)

	African-American (N = 3,022) (MI/Fatal CHD = 403)						White (N = 8,987) (MI/Fatal CHD =933)					
	Model 1			Model 2			Model 1			Model 2		
dbSNP ID	HRR	95 % CI	p	HRR	95 % CI	p	HRR	95 % CI	p	HRR	95 % CI	p
rs2736122*												
CHD	1.20	1.03, 1.40	0.02	1.18	1.01, 1.39	0.04	0.95	0.86, 1.06	0.38	0.94	0.85, 1.05	0.29
rs4246742*												
CHD	1.00	0.87, 1.15	0.97	0.98	0.85, 1.14	0.83	0.96	0.85, 1.09	0.56	0.99	0.87, 1.13	0.88
rs6863494												
CHD	0.93	0.67,1.29	0.66	0.95	0.68, 1.32	0.77	--			--		
rs4975605												
CHD	1.04	0.91, 1.19	0.55	1.05	0.91, 1.20	0.52	1.02	0.94, 1.12	0.61	1.02	0.93, 1.11	0.74
rs2736100												
CHD	0.99	0.86, 1.14	0.89	1.00	0.86, 1.15	0.97	0.97	0.88, 1.06	0.48	0.98	0.89, 1.07	0.60
rs2853668												
CHD	0.93	0.81, 1.07	0.32	0.98	0.84, 1.13	0.73	1.05	0.95, 1.16	0.35	1.07	0.97, 1.19	0.19

dbSNP, The National Center for Biotechnology Information’s SNP database; SNP, single nucleotide polymorphism; ID, identification; N, number; HRR, hazard rate ratio; CI, confidence interval; p, p-value for hazard rate ratios from Cox regression models; MI, myocardial infarction; CHD, coronary heart disease; Model 1, adjusted for age and gender; Model 2, adjusted for age, gender, BMI, current smoking, diabetes, hypertension, HDL cholesterol, and LDL cholesterol; *not consistent with proportional hazards assumptions in whites for rs2736122 (Model 1) and rs4246742 (Models 1 and 2)

**Table 5:** *TERT* sequence variation and incident ischemic stroke. ARIC study (1987 – 2011)

	African-American (N = 3,022) (Ischemic Stroke = 287)						White (N = 8,987 ) (Ischemic Stroke = 452)					
	Model 1			Model 2			Model 1			Model 2		
dbSNP ID	HRR	95 % CI	p	HRR	95 % CI	p	HRR	95 % CI	p	HRR	95 % CI	p
rs2736122												
Isch. stroke	0.89	0.73, 1.08	0.24	0.90	0.74, 1.10	0.32	1.01	0.87, 1.17	0.89	1.02	0.87, 1.18	0.82
rs4246742												
Isch. stroke	0.94	0.79, 1.11	0.47	0.96	0.80, 1.15	0.67	0.99	0.82, 1.18	0.89	1.00	0.83, 1.21	0.99
rs6863494												
Isch. stroke	0.90	0.61, 1.33	0.59	0.96	0.65, 1.42	0.84	--			--		
rs4975605												
Isch. stroke	0.94	0.80, 1.11	0.48	0.88	0.74, 1.04	0.14	1.00	0.88, 1.14	0.97	1.00	0.87, 1.14	0.98
rs2736100												
Isch. stroke	1.07	0.91, 1.27	0.41	1.03	0.86, 1.22	0.75	0.93	0.82, 1.06	0.31	0.93	0.81, 1.06	0.27
rs2853668												
Isch. stroke	1.17	1.00, 1.38	0.05	1.08	0.91, 1.28	0.36	1.02	0.88, 1.18	0.76	1.03	0.89, 1.20	0.71

dbSNP, The National Center for Biotechnology Information’s SNP database; SNP, single nucleotide polymorphism; ID, identification; N, number; HRR, hazard rate ratio; CI, confidence interval; p, p-value for hazard rate ratios from Cox regression models; Model 1, adjusted for age and gender; Model 2, adjusted for age, gender, BMI, current smoking, diabetes, hypertension, HDL cholesterol, LDL cholesterol; Isch., ischemic

**Table 6:** *TERT* sequence variation and all-cause mortality. ARIC study (1987 – 2011)

	African-American (N = 3,319) (Deaths = 1,203)						White (N = 9,827) (Deaths = 2,875)					
	Model 1			Model 2			Model 1			Model 2		
dbSNP ID	HRR	95 % CI	p	HRR	95 % CI	p	HRR	95 % CI	p	HRR	95 % CI	p
rs2736122												
Mortality	1.05	0.95, 1.15	0.36	1.04	0.94, 1.14	0.47	1.04	0.98, 1.10	0.22	1.03	0.97, 1.10	0.27
rs4246742												
Mortality	0.99	0.91, 1.08	0.85	0.99	0.90, 1.08	0.76	1.01	0.94, 1.08	0.80	1.02	0.95, 1.09	0.63
rs6863494												
Mortality	1.00	0.84, 1.20	0.96	1.00	0.84, 1.20	0.96	--			--		
rs4975605												
Mortality	0.96	0.89, 1.04	0.37	0.94	0.87, 1.02	0.16	1.00	0.95, 1.05	0.96	0.99	0.94, 1.04	0.74
rs2736100												
Mortality	1.00	0.92, 1.09	0.93	0.98	0.90, 1.07	0.73	1.03	0.98, 1.09	0.21	1.03	0.97, 1.08	0.32
rs2853668*												
Mortality	1.00	0.92, 1.09	0.94	0.97	0.89, 1.05	0.44	0.99	0.93, 1.05	0.73	1.02	0.96, 1.08	0.62

dbSNP, The National Center for Biotechnology Information’s SNP database; SNP, single nucleotide polymorphism; ID, identification; N, number; HRR, hazard rate ratio; CI, confidence interval; p, p-value for hazard rate ratios from Cox regression models; Model 1, adjusted for age and gender; Model 2, adjusted for age, gender, BMI, current smoking, diabetes, hypertension, HDL cholesterol, LDL cholesterol; * not consistent with proportional hazards assumptions in whites for rs2853668 (Model 1)

## Discussion

A functional role for telomerase in the maintenance of telomere length has been established both *in vitro* and *in vivo,* including in the heart [36]. In an early test of the proposed causal relationship between telomere attrition and cellular senescence, retinal pigment epithelial cells and foreskin fibroblasts that do not normally express telomerase were transfected with the enzyme’s catalytic subunit. The telomerase positive clones exhibited elongated telomeres and exceeded their normal life span by more than 20 cell divisions [37]. Similarly, restoration of telomerase activity in *Terc*-deficient mice resulted in longer telomeres and absence of premature aging [38], and alleviated the tissue degeneration and activation of DNA damage signaling that are characteristic consequences of telomere loss [39]. Forced expression of *TERT* in cardiac muscle in mice promoted cell proliferation and cardiac myocyte survival, suggesting a possible strategy for organ regeneration after injury [40].

Leukocyte telomere length has been shown to be associated with cardiovascular disease risk in some but not all studies [7–17]. In the current study, a nominal association between rs2736122 and incident CHD in the fully adjusted model (HRR = 1.18, p = 0.04, 95 % CI = 1.01– 1.39), and rs2853668 and incident ischemic stroke in a regression model adjusted only for age and gender (HRR = 1.17, p = 0.05, 95 % CI = 1.00 – 1.38) was detected in African-American ARIC study participants. These observations are in accordance with an earlier report that 5 *TERT* SNPs including rs2736100 and rs4975605, and 2 variants including rs2853668 were associated with risk of incident nonfatal MI and ischemic stroke, respectively, in 23,294 individuals of European ancestry enrolled in the Women’s Genome Health Study (WGHS) [41]. However, rs2853668 was associated with a reduced susceptibility to ischemic stroke in the WGHS (HRR (stroke) = 0.81, p = 0.03, 95 % CI = 0.66 – 0.98) after adjustment for age, BMI, smoking, diabetes, hypertension, and hormone use while the same variant increased risk in the ARIC study. Although there was adequate power to detect the same HRR observed by Zee et al., none of the three polymorphisms found to be associated with cardiovascular disease in the WGHS that were also genotyped in the ARIC study (rs2736100, rs4975605, rs2853668) [41] were associated with stroke or CHD in white study participants. Other reasons for the discordant findings could be associations that were found by chance in either or both cohorts, as well as differences in ascertainment since WGHS included only nonfatal MI cases in the analyses while the ARIC study case definition encompassed both MI and fatal CHD.

Although the observed associations between the *TERT* variants and both CHD and stroke were modest in African Americans and were no longer significant after correction for multiple testing, differences in LD could contribute to the absence of an association in whites if a true causative variant was only correlated with rs2736122 or rs285366 in African-Americans. Inspection of the LD plots generated at the *TERT* locus for the Utah residents with Northern and European ancestry (CEU) and African ancestry in Southwest USA (ASW) populations included in the International HapMap Project reveals that the race-specific LD patterns are not identical, with the caveat that this region has not been densely genotyped (HapMap3 Genome Browser release #2, chromosome 5: positions 1,306,287 – 1,348,162) [27]. Similarly, variation in linkage disequilibrium structure between whites and African-Americans could also explain the reversal in the direction of association of rs2853668 with incident ischemic stroke seen in the ARIC study when compared with the WGHS. Assuming that rs2853668 may not be the causal variant in either cohort, correlation between the polymorphism and a protective allele at another locus in WGHS participants, and with a risk allele in ARIC participants could lead to the observed results [42]. Other reasons for the discrepancy could include chance, differences in allele frequency for the rs2853668 T allele in the two racial groups, and variation in other genetic or environmental factors that may contribute to cerebrovascular disease risk in the two study populations. The *TERT* rs2736100 variant was not associated with incident CHD or stroke in either racial group. In the GWA study of telomere length in which rs2736100 was identified, there was also no relationship between this variant and prevalent coronary artery disease in a meta-analysis that combined the results for 22,233 cases and 64,762 controls of European ancestry who were enrolled in the CARDIoGRAM consortium but did not include individuals of African descent [21, 43].

The relationship between telomere length and aging and longevity has also been assessed. A negative correlation between telomere length and age has been consistently observed when examined in multiple tissues [3, 44–47]. More recently, telomere length was positively correlated with increased lifespan in the Amish Family Osteoporosis Study [18], and Fitzpatrick et al. reported that individuals in the shortest quartile of leukocyte telomere length in the Cardiovascular Health Study were more likely to die than those in the longest quartile during a 6.1-year follow-up period [20]. In contrast, Bischoff et al. found no correlation between telomere length and survival in a sample of 812 individuals from 3 different Danish study populations [48]. Similar results were reported in the Scottish Lothian Birth Cohort [49], and in a study of 3,075 participants in the population-based Health ABC Study aged 70–79 years in which neither overall survival or death from cardiovascular disease was associated with telomere length [50]. While an association between two polymorphisms in oligonucleotide/oligosaccharide-binding fold containing 1(*OBFC1)*, a gene related to telomere length [51], and decreased risk of cardiovascular death was demonstrated in women in the Cardiovascular Health Study [52], none of the *TERT* sequence variants examined here had a discernible effect on the time to death in ARIC study participants.

For all of the statistical analyses described above, it is possible that, although there was only a marginal effect on the risk of developing cardiovascular disease when the *TERT* polymorphisms were considered individually, the polymorphisms may play a role in combination with other loci associated with variation in telomere length as demonstrated by Codd et al. in a genetic risk score analysis for coronary artery disease [21]. In addition, since the association between the *TERT* polymorphisms and telomere length could not be evaluated in the ARIC study, a link between increased risk of cardiovascular disease and the possible functional impact of the gene could not be explored further. It should also be noted that since several risk factors for cardiovascular disease including obesity and smoking have been shown to be associated with telomere length in leukocytes [53], differences in the distribution of these covariates between populations or racial and ethnic groups could result in inconsistencies in the reported relationship between *TERT* and a given disease outcome. Further investigation of sequence variation in *TERC* [54] as well as other genes such as *OBFC1*, CTS telomere maintenance complex component 1 (*CTC1*), and zinc finger protein 676 (*ZNF676*) that have been identified and replicated in large-scale GWA studies of telomere length [51, 55] but were not present on the genotyping array may also prove to be informative in the ARIC cohort.

## Conclusions

The association between six *TERT* polymorphisms that tag the variation in this gene and development of MI and ischemic stroke over a 20-year follow-up period was examined in white and African-American ARIC study participants with no prior history of disease. After adjustment for age and gender, rs2736122 and rs2853668 were nominally associated with incident CHD and stroke, respectively, in African-Americans but not in whites. The results suggest that interindividual variation in a gene implicated in cellular aging may be associated with cardiovascular disease, and that replication in other population-based cohort studies is warranted.

## Abbreviations

ARIC: Atherosclerosis Risk in Communities
BMI: Body mass index
CARe: Candidate Gene Association Resource
CHD: Coronary heart disease
GWA: Genome-wide association
HDL: High density lipoprotein
HRR: Hazard rate ratio
IBC: ITMAT-Broad-CARe
LD: Linkage disequilibrium
LDL: Low density lipoprotein
MI: Myocardial infarction
OBFC1: Oligonucleotide/oligosaccharide-binding fold containing 1
SNP: Single nucleotide polymorphism
TERC: Telomerase RNA component
TERT: Telomerase reverse transcriptase
WGHS: Women’s Genome Health Study
